# Constraints and restraints in crystal structure analysis

**DOI:** 10.1107/S0021889808044142

**Published:** 2009-02-07

**Authors:** Attilio Immirzi

**Affiliations:** a Dipartimento di Chimica, Università di Salerno, I-84084 Fisciano (SA), Italy

**Keywords:** constraints, restraints, crystal structure analysis

## Abstract

The widely used restraint-based approach to structural analysis using diffraction data is critiqued. The convenience of using rigid constraints, through the use of internal coordinates, is discussed.

## Abstract 2

The restraint-based procedure in least-squares refinement is critiqued and the advantages of using internal coordinates are discussed.

## 1. Introduction

The aim of this letter is to express criticism of the widely used restraint-based approach to structural analysis from diffraction data and to underline the advantages of using constraints and internal coordinates. The debate is decidedly aged, but I believe that reopening it is desirable.

The problem of how to perform least-squares (LS) structural refinement from X-ray diffraction measurements, taking into account the subsidiary structural information available (known bond lengths, bond angles *etc.*), was debated back in the 1960s. The vexed question was whether to use constraints (precise specifications) or restraints (flexible specifications).

Constraints have, in fact, been used sparingly in the past 40–50 years (see later); restraints, on the other hand, have been used abundantly and are still widely employed. Another aim of this letter is to give reasons for the low popularity of constraint-based methods. 

The ordinary LS procedure involves finding the minimum of the sum 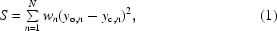
where 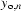 are 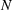 measurable values and 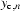 the corresponding values computable as functions of 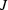 variables 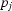, with 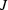 much less than 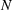, and where 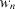 are appropriate weight factors. In diffraction analysis, 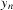 are either the squared structure factors 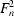 or the moduli 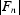; the variables 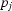 are structural variables, commonly the atomic fractional coordinates (a.f.c.).

Countless coordinate systems can be used, of course, as alternatives to the a.f.c., provided there are biunivocal relationships. The convenience of using internal coordinates (i.c.) for defining the molecular structure (bond lengths, bond angles and torsion angles) was soon recognized (see *e.g.* Wilson *et al.*, 1955 ▶), since chemically connected atoms frequently have foreseeable distances and/or angles. Even so, other parameters will be necessary for defining the position and orientation of the molecules in the crystal, *viz*. molecular rotations and translations.

The number of i.c. needed for modelling an 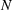-atom crystal structure is 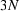, the same as the a.f.c. In the case of molecular crystals without symmetry, there are six rigid-body parameters and 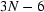 molecular i.c. to be assigned among interatomic distances and angles. The latter are indeed more than 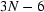 and the selection of i.c. among bond lengths and angles must be done carefully to produce a non-redundant coordinate system (Califano, 1974 ▶; Pulay *et al.*, 1979 ▶). There are several good reasons for pursuing non-redundance. The first is that, in modelling molecular structures, non-redundant i.c. behave as strictly independent variables, so that the a.f.c. are analytical functions of the i.c., whilst redundant coordinates imply non-analytical building steps (*e.g.* solving one or more equations); the second reason is that, in performing LS refinements, the number of degrees of freedom is reduced and one obtains matrices of the smallest possible size; the third is that redundant systems imply singular matrices and matrix inversion with standard procedures (*e.g.* Gauss-Jordan & Cholesky; see Press, 1996 ▶) is not allowed.

Finally, if redundancy is avoided, carrying out a structural refinement based on internal coordinates is as simple as in the a.f.c. case, with the added advantage of having a smaller number of variables, if a number of i.c. (typically bond lengths, but also bond angles in certain cases) can be kept fixed. This applies of course in difficult cases, with a low data-to-unknown ratio. As discussed elsewhere (see Immirzi, 2007*b* ▶), there is a general rule, which is applicable to all molecular crystals, for choosing the i.c. correctly: include all bond lengths among the i.c., then choose the other i.c. among angles, considering carefully the kind of construction adopted. There are several possibilities, the best known being the *z*-matrix method, devised by Eyring (1932 ▶). Other methods, discussed elsewhere (Immirzi, 2007*a* ▶,*b* ▶), make up for the limitations of the *z*-matrix method, which is not sufficient to cover all cases.

## 2. Using constraints and internal coordinates

If subsidiary information is available that can be considered as precise specifications, which analytically assume the form of 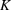 equations (constraints) of type 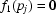, 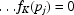, one could find the above minimum of 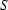 [equation (1)], whatever the coordinate system is, by adopting the method devised by Lagrange (1797 ▶), later termed the method of the undeterminate multipliers (Mellor, 1912 ▶). Hughes (1941 ▶) discussed the method in the crystallographic context; Waser (1963 ▶) pointed out that the method, while elegant, is often cumbersome in numerical applications. The problem is that when dealing with 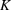 constraints, the above LS matrix is not 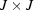 but 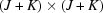 instead. As a consequence, the matrix becomes, when 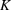 is more than a few units, rather ill conditioned and the procedure impractical.

Only a few crystallographic problems can be treated using the Lagrange method; one is the chain continuity in polymers (see Tadokoro, 1979 ▶; Immirzi *et al.*, 2007 ▶). If precise specifications are numerous, it is decidedly better to use i.c. instead of ordinary a.f.c. With this strategy, the size of the LS matrix does not increase but decrease, since the number of i.c. truly optimized is much less than the number of the a.f.c.

The internal coordinates route, without deepening the redundancy problem (see above), was followed by Arnott & Wonacott (1966 ▶) who implemented the well known computer program *LALS* (linked atoms least squares; see also Smith & Arnott, 1978 ▶), which is of general applicability but has been used mainly in polymer crystallography. *LALS* has been repeatedly updated; the latest version is that reported by Okada *et al.* (2003 ▶).

There are other computer programs (*e.g.* *SHELXL*; Sheldrick, 2008 ▶) claiming the use of constraints without using internal coordinates, however. Such a procedure is limited to the case of linear relationships between a.f.c. accounted for by appropriate elimination of one variable computed as a function of others. To give a simple example, an atom lying along the 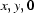 diagonal (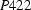 space group) is ‘constrained’ to have 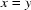 and this identity can be imposed with this machinery. There are, however, many more complicated situations (*e.g.* local non-crystallographic symmetries) and an elegant general solution exists for treating them with simplicity: using i.c. and using a symbolic language for the molecular modelling. The general-purpose program *TRY*, available free of charge on the Web (http://www.theochem.unisa.it/try.html), allows this.

## 3. Using restraints

On the whole, constraints are used somewhat infrequently. In contrast, restraints are used rather liberally by most crystallographers whenever they are dealing with many variables and limited data, and also simply when they are dissatisfied with the results obtained with the ordinary procedure. Studies done using restraints are very numerous; in protein crystallography they are used very extensively.

The restraint-based least-squares approach was first proposed by Waser (1963 ▶); recent articles have been written by Watkin (1994 ▶) and by Prince *et al.* (1999 ▶). The well known crystallographic package *SHELX* (Sheldrick, 2008 ▶) also makes use of restraints. Waser’s idea was to add to the above sum 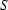 [equation (1)] a second sum 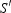 to be performed on a number of quantities 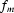 (typically bond lengths and bond angles) also computable as a function of the structural variables and having target values 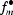: 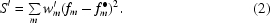
The minimum of 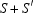 is pursued instead of 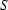. In practice, the role of 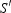 is that of ‘forcing’ the 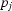 variables towards values rendering 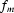 close to 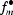. The 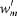 are appropriate weights assigned by the user; the higher the 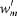, the stronger the forcing. The ‘idealized’ values for 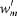 would be 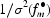, the latter being the standard deviations for the 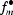 observed in the reference structural models. Frequently, 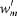 are more or less arbitrary.

## 4. Critical observations

In the author’s opinion, the restraint-based LS procedure, although founded on heuristic considerations, needs to be questioned. Waser’s idea of treating the subsidiary conditions as if they were ‘observational equations’ is wanting, because it regards experimental data (the 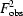 values) as analogous to the subsidiary information, whereas in fact the latter are merely ‘expectations’. Summing data and expectations in constructing the LS matrix brings about several nonsensical inconsistencies.

Arguments against the restraint-based LS fit are as follows.

(1) The sums 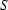 [equation (1)] and 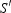 [equation (2)] are intrinsically non-homogeneous, even if the summed items are rendered adimensional in both cases by defining the 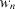 and 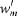 properly. Note that 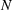 (the number of observations) can be as high as one wishes, without an upper limit, and the measurement of each 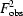 may be repeated many times; 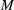 (the number of restrained quantities) depends instead on the actual molecular structure and cannot be increased or decreased arbitrarily. Consequently, in the computation of the LS matrix, the role of 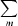 can be arbitrarily reduced or enhanced and the same applies to the parameter shifts.

(2) It was stated earlier that Waser’s idea is applicable whatever the cordinates are, and applies also to the internal coordinates themselves. Thus, let us perform two computations, both based on an appropriate set of i.c.: in the first case, perform a regular LS cycle without restraints, refining all the 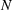 i.c. which, being initially 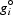, become 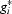; in the second case, refine the first 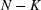 i.c., apply 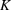 restraints to the last 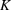 i.c., and use as ‘target’ just the values 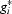. This second computation (the number of degrees of freedom reduces from 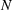 to 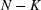) will bring the i.c. to 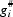, which in general are different from 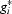. If high 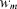 values are used (at the limit infinity) and small 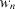 values (at the limit zero), the i.c. for the last 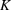 terms will coincide with 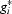 (as target), but the first 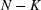 i.c. will gain totally random values since the constructed LS matrix does not depend in any way on the measurement performed. It is evident that this is nonsense: the procedure, any mathematical procedure, must obey the continuity requirements.

(3) The convergence test (the ability of the procedure to find a solution after a number of cycles), a fundamental criterion for evaluating the reliability of the structural model in an LS fit, may become meaningless when the 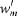 weights are high. Indeed, convergence always takes place, provided the 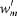 weights are large enough.

(4) A multivariate regression finds a minimum moving in a multi-dimensional space. If no restrictions apply, the minimum point may be everywhere; if one restriction applies, the minimum point is compelled onto a manifold, and if there are more restrictions, it is compelled onto the intersections of many manifolds. Using restraints, therefore, is like simply moving in the vicinity, and it is a very complicated and risky affair! Turning to internal coordinates (cleverly chosen), and refining only the i.c. truly unknown, one reduces drastically the dimensionality of the space and chases a point without any restriction, forgetting manifolds and other mathematical devilries. Why run along tortuous routes when you can follow straight ones?

Finally, it is worth noting that, in using restraints, the number of refined parameters and the size of the LS matrices are the same as if no restraints were imposed. By contrast, constraint-based methods imply a robust reduction in the number of parameters to be adjusted and, consequently, the reliability of the fit, the convergence *etc.* are considerably improved. In special cases, the reduction may be drastic. To give an impressive example, an unsymmetric calix[6]arene (54 atoms) has 162 a.f.c. and can be modelled, at fixed bond lengths, using only 12 angles (see the *TRY* user manual). When the conditions are difficult (many unknowns and limited data) the advantages are evident.

## 5. Concluding remarks

This letter does not set out to dismiss the restraint-based LS approach proposed by Waser as wrong, only to point out that there are wobbly foundations and that there is a risk, especially when restraints are overused, of incorrect results. In contrast, the constraint-based LS approach is unexceptionable when internal coordinates are properly chosen. This should be enough to reopen a critical discussion on the rather outmoded (and hastily archived) question of whether it is better to use constraints or restraints when dealing with complicated molecules. In the author’s opinion, the constraints route is decidedly preferable when there are many parameters and limited data. If, instead, the situation is the reverse, both constraints and restraints are superfluous instruments. Although they do not often cause trouble, it is preferable not to use them.

In the author’s opinion, the poor uptake of constraint-based refinements in X-ray structural analysis, necessarily based on internal coordinates and not on atomic coordinates, is due to a general disregard of the fundamental point discussed above: the necessity of using non-redundant coordinate systems. In addition, computer programming is difficult if one wishes to create systems of general validity. By contrast, the fortunes of the restraint-based method were mainly a consequence of the procedural simplicity and the relatively simple programming.

## References

[bb1] Arnott, S. & Wonacott, A. J. (1966). *Polymer*, **7**, 157–166.

[bb2] Califano, S. (1974). *Vibrational States*, p. 275. New York: J. Wiley and Sons.

[bb3] Eyring, H. (1932). *Phys. Rev.* **39**, 746–748.

[bb5] Hughes, E. W. (1941). *J. Am. Chem. Soc.* **63**, 1737–1752.

[bb7] Immirzi, A. (2007*a*). *J. Chem. Inf. Model.* **47**, 2263–2265. 10.1021/ci700225x 17973364

[bb8] Immirzi, A. (2007*b*). *J. Appl. Cryst.* **40**, 1044–1049.

[bb6] Immirzi, A., Alfano, D. & Tedesco, C. (2007). *J. Appl. Cryst.* **40**, 10–15.

[bb9] Lagrange, J. L. (1797). *Théorie des Fonctions Analytiques*, p. 198. Paris: Imprimerie de la République.

[bb11] Mellor, J. W. (1912). *Higher Mathematics for Students of Chemistry and Physics*, edited by J. A. Landau. London: Longman.

[bb12] Okada, K., Noguchi, K., Okuyama, K. & Arnott, S. (2003). *Comput. Biol. Chem.* **27**, 265–285. 10.1016/s0097-8485(02)00076-1 12927102

[bb14] Press, W. H., Teukolsky, S. A., Vetterling, W. T. & Flannery, B. P. (1996). *Numerical Recipes in Fortran 77*, 2nd ed. Cambridge, New York: Cambridge University Press.

[bb15] Prince, E., Finger, L. W. & Konnert, J. H. (1999). *International Tables for X-ray Crystallography*, Vol. C, edited by E. Prince, pp. 693–701. Dordrecht: Kluwer Academic Publishers.

[bb13] Pulay, P., Fogarasi, G., Pang, F. & Boggs, J. E. (1979). *J. Am. Chem. Soc.* **101**, 2550–2560.

[bb16] Sheldrick, G. M. (2008). *Acta Cryst.* A**64**, 112–122. 10.1107/S0108767307043930 18156677

[bb17] Smith, P. J. C. & Arnott, S. (1978). *Acta Cryst.* A**34**, 3–11.

[bb18] Tadokoro, H. (1979). *Structure of Crystalline Polymers* New York: J. Wiley and Sons.

[bb19] Waser, J. (1963). *Acta Cryst.* **16**, 1091–1094.

[bb20] Watkin, D. (1994). *Acta Cryst.* A**50**, 411–437.

[bb21] Wilson, E. B., Decius, J. C. & Cross, P. C. (1955). *Molecular Vibrations* New York: McGraw Hill.

